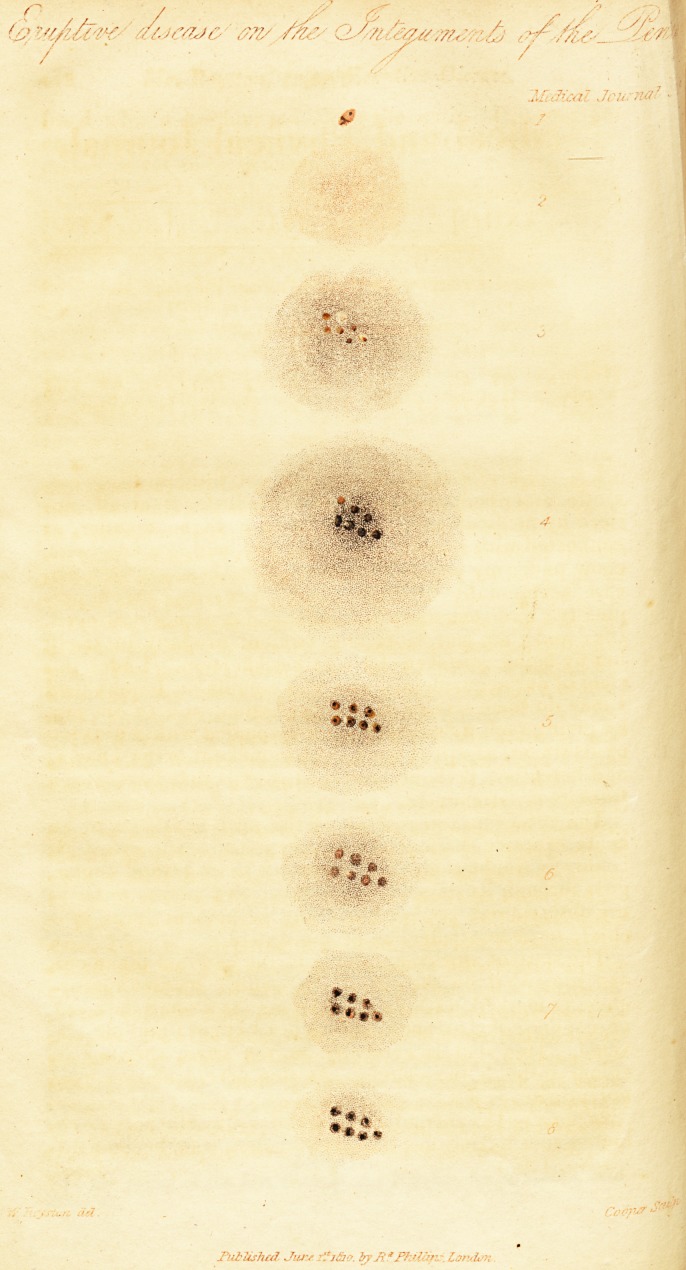# History of an Acute Eruptive Disease of the Integuments of the Penis

**Published:** 1810-06

**Authors:** 


### Medical and Physical Journal.

VOL. XXIII.] JUNE, 1810. [NO. 136.

*Printed for R. PHILLIPS, by W. Thorne, Red Lion Court, Fleet Street, London.*

### HISTORY OF AN ACUTE ERUPTIVE DISEASE OF THE INTEGUMENTS OF THE PENIS. By Mr. ROYSTON.

( *With a Plate.* )

AN eruptive disease, which has not hitherto been correctly described, and, perhaps, very little, if at all, noticed by medical writers, except in a vague manner, as connected with *syphilis*, has frequently, within a few years, come under my notice. Though slight in itself, this disease accidentally becomes serious, occasions much perplexity; and sometimes has endangered, if not destroyed, life: and that, not from any virulent property in itself, but by being mistaken for another affection. Under this state of our acquaintance with this complaint, I apprehend I may do some service to the profession, and to society, by giving such a description, accompanied with a drawing of its stages, as will at least, tend to make it better known; and hereafter, perhaps, prevent the evils that have arisen from its mal-treatment.

The cutaneous integument of the *penis* is subject to an acute eruptive disease of no infrequent occurrence. It is always troublesome, but when left to its natural course, goes through its stages in eight days; and never, as far as my observations go, produces any permanent local mischief, or general affection of the system.

The scite of this complaint is in the external tegument, and somewhat on the side of the penis. Its first appearance, as marked, (1.) on the drawing, is a small dark red, or copper-coloured spot, with defined edges; and not elevated. This spot is evidently in the cutis, shewing its colour through the cuticle. At this period neither pain nor pruritus is felt.

*2d day.*—The second stage is marked by the copper-coloured spot having lost its defined edges, and become diffused with a brighter red along the cutis. It is now accompanied with a slight pruritus, but no elevation of the cuticle is yet to be detected.

*3d day.*—On the third day, the inflammation is further spread, the pruritus is increased; and in the centre of the inflamed disc several, usually from six to ten, lucid vesicles may be observed. These vesicles contain a transparent lymph, through which the colour of the inflamed cutis is discerned.

*4th day.*—All the symptoms are increased on the fourth day; the pruritus becomes troublesome, and some tumefaction of the integument of the penis is manifest. The vesicles are enlarged, more elevated, irregular in shape, and have a tendency to become confluent. But they yet retain their lucid transparency.

*5th day.*—On the fifth day, the inflammation, pruritus, and tumefaction, in a degree, subside. The vesicles are now becoming opake, by the contained fluid approaching to a purulent form. At this period, the cuticle is apt to be ruptured by some accident; and on the contents of the vesicles being removed, the cutis is found, under each of them, in a state of ulceration.

*6th day.*—The inflammation still subsiding, the vesicles become completely opake, and contain a distinctly formed pus. The pruritus, which had begun to decrease on the fifth day, has now vanished, but is succeeded by a tenderness; and touching the part gives pain.

*7th day.*—An approach to the healthy state is now manifested by the removal of all irritation, and by the vesicles assuming a brownish hue.

*8th day.*—On this day the only remaining mark of disease is a hard, elevated, brown substance, (the purulent discharge indurated) adhering firmly to the cutis, where each vesicle had been. The removal of this substance brings into view a decided vestige of previous ulceration.

The remote or proximate cause of this well-marked disease, I am not prepared to explain. It has been suggested to me by a gentleman, extremely conversant in the causes of febrile affections, who is a close and attentive observer, and has made considerable researches into cutaneous diseases, that the local complaint here described is always preceded by some, though slight, *pyrexia.* Rigours, lassitude, and the usual symptoms of the attack of exanthemata, in a lower degree, or proportionate to the extent of the local affection, he is of opinion, always precede this eruption. This, however, I have never yet perceived. On the the contrary, my observations have led me to remark, that it has frequently occurred in a state of perfect general health: that during its progress, no other derangement of the system has arisen, than what might be fairly attributed to mental perturbation.

As the first appearance of this complaint, marked (1) in the drawing, has frequently happened within twenty-four hours after the sexual intercourse, it was no forced presumption, that some circumstance of that act produced it: and this opinion is strongly supported by the fact of those who live in a state of abstinence, in that particular, being free from it. But whether, if it is thus produced, it arises directly from some acrimony in the female secretions; indirectly from mechanical action, applied to parts predisposed to receive such an impression; or is an effect produced by the conjunct operation of both these causes, I am not in possession of facts to determine. I am, however, persuaded, that no abrasion of the cuticle precedes the attack of this disease; and it can hardly be believed, that if it arose from the application of any acrimony *ab extra*, that in no instance this acrimony would act upon the more susceptible parts about the glans and inner portion of the prepuce; but always upon that part where the cuticle presents a firmer defence. These observations call up the question of its infectious or non-infectious nature. If it is excited by the application of some acrimonious fluid, communicated by the female to the male, the possibility of reciprocal excitement will not be denied. This I had long doubted; but a recent case has convinced me, that such a communication of the disease does really exist. On the fifth day of the complaint, when it was evidently subsiding, the application of the purulent discharge to the labia and nymphæ excited considerable inflammation, intolerable pruritus and tumefaction, but without any evident vesication.

The effect of this stimulus, whatever it is, appeared in twenty four hours after its application; continued to increase to the end of the fourth day, and then gradually declined.

The affinity between this and other acute diseases of the skin is characterized by two circumstances. In the regularity of its progress, it strikingly resembles the *exanthemata*; and its stages, when undisturbed by art, are as decidedly marked as those of distinct *variola*. Its external appearance has the character of the genus erysipelas, especially resembling in the early part, (third and fourth day) of its vesicular stage, the *zona ignea*: with this difference, however, that the cuticle does not spontaneously burst, (vide fifth day) and permit the contained fluid to escape; for, if not accidently ruptured, it remains whole to the last period, when the appearance described in the 8th day takes place.

Opinions found in authors are false, though harmless, when they pronounce this disease to be an excoriation. When it has been taken for a venereal affection, and this has not unfrequently happened, the consequences have sometimes been very serious. The evening of the 4th, or the morning of the 5th day, is the usual time when professional assistance is required. At this period, the ulcerated state of the cutis (for the cuticle has commonly been removed by some lotion, or washed away with a view to cleanliness) gives it, to a hasty observer, the appearance of *chancre*. Its recent state, and its presumed locality, seem to justify the immediate application of some escharotic, with the intention suddenly to produce a slough, which will, on its separation, bring away the supposed virus. The inflammation and irritable state of the parts are greatly increased by this process; considerable ulceration ensues; it is thought right speedily to fill the system with mercury: the local disease increases, a febrile state is induced, and kept up partly by mental alarm, and partly by the stimulus of the specific, until a mild morbid action degenerates into a phagedenic sore. Its supposed syphilitic origin countenances a continued use of mercury, until the prepuce and great part of the integuments of the penis are sacrificed. After some weeks, or months of trial, it is discovered that, from the patient's peculiar and undefined idiosyncracy, mercury will not exert its specific properties on the disease. In this deplorable state, *conium* commonly, sometimes *hyoscyamus*, and more rarely, the *atropa belladonna*, are resorted to. This does not unfrequently happen. I have had the chance to see it more than once, and most surgeons who have practiced a few years in the metropolis, have either themselves seen, or learned *a part of its history* from their friends.

When this disease has proceeded to this point, I cannot hesitate to say, that the only remaining chance to save the patient, is that of having recourse to mild and soothing topical applications; by removing him, if in town, to a country air; by the employment of an un-irritating restorative diet; and the use of pleasant tonics. Mercury, and the whole tribe of narcotic vegetables, with the exception ception of opium, if the patient has been accustomed to its use, should be discarded at once.

If the course of this disease has been disturbed by the interference of art, it becomes extremely difficult to determine its nature; and a painful state of perplexity ensues to the surgeon, who sees it under these circumstances. The wavering between its syphilitic origin, and its having some other, (but hitherto) obscure source, is usually determined however, in favour of the former; and precisely that method which creates the danger is pursued, under the opinion of its being most safe. Four years ago, a case of this kind came under my care, at a period when, I confess, I had no clear idea of its history. On the 3d day of the patient's noticing it, but on the 5th from its actual commencement, it was shewn to me. It had then arrived at its purulent stage, but from the cuticle having been removed, there appeared on the integuments, in the usual place of the disease, three small ulcers, accompanied with slight irritation; having in them a character that gave me the opinion that they were slowly enlarging, and possessed some quality different from simple ulceration. The history given by the patient was, that forty-eight hours after having exposed himself to the hazard of contracting syphilis, itching and inflammation had arisen in this part, accompanied with some shining red pimples, and ending in the ulceration I saw. This account, supported by an appearance in the ulcers, though not in itself very decided, led to a conclusion that the safest determination would be to admit the syphilitic origin. It was, perhaps, fortunate for this patient that he had recently suffered by a mercurial course, pushed too far; and that his habit still continued so sensible to the action of that mineral, that the hazard of suddenly filling his system with it, was greater than would arise from the chance of absorption from the ulcers. The mildest escharotics were applied, the usual precautions were taken to avoid, as much as possible, the occasion of irritation, and in four days the ulceration was considerably enlarged. Three weeks elapsed under the application of various preparations of mercury, in ointments and in lotions; and the disease had much increased. The three small ulcers had run into one, making a sore the third of an inch in diameter; foul in its appearance, and slightly inflamed. At this time, a surgeon of great skill and known candour, saw it. His opinion was against its being syphilitic, and advised to have the ulcer dressed twice a day with a solution of nitrate of silver. Thus, occasionally

 varying varying the applications, three weeks more passed away, and the ulcer had evidently increased in size, and its appearance was not improved. During all this time the patient had not taken mercury in any form, for the reason before assigned. In this state of perplexity, it was determined to give one week's trial to the simplest application that could be devised. All the former medicaments were left off, the parts were washed perfectly clean from their remains, and the sore was covered with gold-beater's skin. Twice a day a tepid bath of water was used, after which, fresh gold-beater's skin was applied, and in one week the ulcer was completely healed. This case is a forcible example of a mild disease, which regularly goes through all its stages in a few days, when left to itself, becoming very troublesome by the use of improper topical applications only. Had the patient's constitution been in a state to have admitted of a free use of mercury, it would have been employed in its fullest extent, the result of which would have been the increase of the disease to a very dangerous degree.

As my intention has gone no further than to give, as distinctly as my observations on it permitted, a history of this disease from nature, and connected with the consequences of mistaking it; I have rather avoided than sought the descriptions of it found in books, if any such exist: for I doubt, very much, if the hydatids and crystaline bladders, described in the old authors on lues, as symptoms of that disease, do apply to this. It is but an act of justice to state, however, that the author of the treatise on "Morbid Poisons" has, in that work, observed, "that the crystalines of these authors are only to be considered as a species of epidemic erysipelas, prevalent at a particular season, and which has occasionally occurred since, especially in fleets and armies." This is a step towards the truth. But when epidemic erysipelas attacks this part of the frame, which, I believe, seldom happens, its character will be so strongly marked, and the general derangement of the system so palpable, as scarcely to admit of being mistaken for any other disease, especially syphilis. Whereas the disease I have described, alway arises under suspicious circumstances, occurs at all seasons, and seems independent on their influence; happens generally in the society of civil life, is so perfectly local, never, as far as I know, spreading from the inflamed spot where it arises; and in every instance I have seen, so obviously unconnected with any febrile state of the habit, that the younger branches of the profession, to whom application on on these occasions is commonly first made, will always be liable to mistake it for chancre. And when altered in its appearance, by attempts made to cure it, even the experienced surgeon will, I know, have doubts; and feel that the nature of the case is of difficult determination.

In the treatment of a disease so mild in its symptoms as this, not much is required of the practitioner. The great object is, to have so clear a knowledge of it as never to mistake it for syphilis, to avoid the application of all irritating substances, and to quiet the patient's mind. The topical application which I have found most convenient, has been gold-beater's skin. As soon as the eruption appears, a slip of this substance may be spread over it from time to time. And this, with occasional ablution with tepid water, will comprehend the whole of the curative process. I can suppose, however, that anomalous cases may arise, but which I have not yet seen, where the more active methods to restrain inflammation, or remove its consequences, may be required.

If this disease has hitherto, as I believe, been unnoticed, except in an indecisive manner, more calculated to mislead than instruct, I may have a reasonable hope that this account of it may be of service; and that others will be induced to carry the inquiry further, and render its history so clear, that it will no longer have the chance of being mistaken for syphilis, and rendered formidable by improper treatment.

P. S. It has given me considerable satisfaction to observe, that an inquiry, originally suggested by me, in "Hints for a Medical Topography of Great Britain," (Medical and Physical Journal, January and February 1809) has produced some curious facts in the history of the properties of ipecacuanha. Dr. Hamilton, (Medical and Physical Journal, April 1810, p. 318), says, the case related by Mr. Spencer, of a peculiar property in this plant, appears to be the *first* on the subject. I beg to refer him to the Medical and Physical Journal, (January 1809, p. 20) where he will find the *first* reference to this property of ipecacuanha, and which recalled to Mr. Spencer, the recollection of his apprentice's case. (Medical and Physical Journal, June 1809, p. 485.) The nature of my communication on Medical Topography did not admit of a minute detail of cases, but I mentioned, as concisely as possible, singular facts which had fallen within my own knowledge, with a view to excite inquiry.

*Princes Street, Cavendish Square, May 10, 1810.*

## Figures and Tables

**Figure f1:**